# Formulating Resveratrol and Melatonin Self-Nanoemulsifying Drug Delivery Systems (SNEDDS) for Ocular Administration Using Design of Experiments

**DOI:** 10.3390/pharmaceutics16010125

**Published:** 2024-01-18

**Authors:** Elide Zingale, Angela Bonaccorso, Agata Grazia D’Amico, Rosamaria Lombardo, Velia D’Agata, Jarkko Rautio, Rosario Pignatello

**Affiliations:** 1 Laboratory of Drug Delivery Technology, Department of Drug and Health Sciences, University of Catania, Viale A. Doria 6, 95125 Catania, Italy; elide.zingale@phd.unict.it (E.Z.); angela.bonaccorso@unict.it (A.B.); rosamaria-lombardo@libero.it (R.L.); 2 NANOMED—Research Centre for Nanomedicine and Pharmaceutical Nanotechnology, Department of Drug and Health Sciences, University of Catania, 95125 Catania, Italy; 3 Department of Drug and Health Sciences, Section of Systems Biology, University of Catania, Viale A. Doria 6, 95125 Catania, Italy; agata.damico@unict.it; 4 Department of Biomedical and Biotechnological Sciences, Section of Anatomy, Histology and Movement Sciences, University of Catania, 95100 Catania, Italy; vdagata@unict.it; 5 School of Pharmacy, University of Eastern Finland, Yliopistonranta 1C, 70210 Kuopio, Finland; jarkko.rautio@uef.fi

**Keywords:** SNEDDS, ocular delivery, SIRT-1, stability, experimental design, surfactants, oils

## Abstract

Recent studies have demonstrated that Sirtuin-1 (SIRT-1)-activating molecules exert a protective role in degenerative ocular diseases. However, these molecules hardly reach the back of the eye due to poor solubility in aqueous environments and low bioavailability after topical application on the eye’s surface. Such hindrances, combined with stability issues, call for the need for innovative delivery strategies. Within this context, the development of self-nanoemulsifying drug delivery systems (SNEDDS) for SIRT-1 delivery can represent a promising approach. The aim of the work was to design and optimize SNEDDS for the ocular delivery of two natural SIRT-1 agonists, resveratrol (RSV) and melatonin (MEL), with potential implications for treating diabetic retinopathy. Pre-formulation studies were performed by a Design of Experiment (DoE) approach to construct the ternary phase diagram. The optimization phase was carried out using Response Surface Methodology (RSM). Four types of SNEDDS consisting of different surfactants (Tween^®^ 80, Tween^®^ 20, Solutol^®^ HS15, and Cremophor^®^ EL) were optimized to achieve the best physico-chemical parameters for ocular application. Stability tests indicated that SNEDDS produced with Tween^®^ 80 was the formulation that best preserved the stability of molecules, and so it was, therefore, selected for further technological studies. The optimized formulation was prepared with Capryol^®^ PGMC, Tween^®^ 80, and Transcutol^®^ P and loaded with RSV or MEL. The SNEDDS were evaluated for other parameters, such as the mean size (found to be ˂50 nm), size homogeneity (PDI < 0.2), emulsion time (around 40 s), transparency, drug content (>90%), mucoadhesion strength, in vitro drug release, pH and osmolarity, stability to dilution, and cloud point. Finally, an in vitro evaluation was performed on a rabbit corneal epithelial cell line (SIRC) to assess their cytocompatibility. The overall results suggest that SNEDDS can be used as promising nanocarriers for the ocular drug delivery of RSV and MEL.

## 1. Introduction

Many degenerative ocular diseases such as cataracts, macular degeneration, diabetic retinopathy (DR), glaucoma, and optic neuritis are associated with a downregulation of Sirtuin-1 (SIRT-1) [1,2,3,4,5,6]. Looking specifically at posterior eye diseases, the role of SIRT-1 in DR has been extensively studied, but the complex molecular interactions are not fully understood. However, in recent years, numerous studies have demonstrated a strong link between SIRT-1 expression and the development of DR. In advanced pathological conditions, hyperglycemia lowers intracellular NAD+ levels and reduces SIRT-1 expression. SIRT-1 is a histone deacetylase protein involved in numerous pathways related to inflammation and oxidative stress. An over-repression of SIRT-1 can suppress inflammation in various tissues, whereas its deletion causes an increase in inflammation locally. By acting on the mediators of inflammation, e.g., through the suppression of NF-kB, SIRT-1 can modulate and reduce the inflammatory response [2]. Indeed, it becomes a therapeutic target for oxidative stress-associated diseases that have been extensively studied. Among them, DR, age-related macular degeneration (AMD), and glaucoma are the most studied conditions in relation to the downregulation of this enzyme. 

Some natural compounds have been shown to be potent activators of SIRT-1, inducing beneficial effects that demonstrate SIRT-1 as a potential target against the inflammation process [7]. For instance, resveratrol (RVS) is able to activate SIRT-1 allosterically; however, its clinical utility is compromised by poor water solubility, instability, and scarce bioavailability [8,9]. RSV protects the ocular tissues from degeneration, including the retinal tissue, by promoting the SIRT-1 pathway [10,11]. Melatonin (MEL) is another excellent regulator of SIRT-1 as its administration induces an upregulation of the enzyme levels. Its role as a modulator of SIRT-1 has been observed in tissues in the testes, ovaries, heart, and nervous system [12,13,14,15]. 

MEL activity on SIRT-1 has also been linked to protection at an ocular level. MEL supplementation demonstrated a protective effect on the retina in an elderly diabetic rat model. The protective effect of MEL supplementation occurs by increasing both retinal antioxidant activity and retinal SIRT-1 gene expression [16]. Most treatments for ocular disorders involve the oral administration of MEL. Its low bioavailability, however, distresses its therapeutic efficacy. Only a low amount of the administered drug reaches the target sites; thus, larger doses and repeated administrations are required.

In this context, discussing nanotechnological systems to improve the efficacy of these two molecules, as well as other natural SIRT-1 agonists, appears to be an innovative approach. Among the latest generation of colloidal carriers, in situ nanoemulsifying systems (SNEDDS) seem to be highly promising but still insufficiently studied. No studies in the literature concern the development of SNEDDS for delivering SIRT-1 agonists into the eye. SNEDDS can be considered an “advanced” formulation compared to micro- and nanoemulsions since they are emulsified directly in situ, avoiding drug loss during storage [17]. SNEDDS are very simple formulations consisting of three components in an anhydrous mixture: oil, surfactant, and co-surfactant (or co-solvent). When considering ocular delivery, with the known technological constraints outlined by pharmacopeias, the choice of the starting materials and the construction of a ternary plot are crucial factors for obtaining formulations appropriate for an industrial scale-up [18,19]. The present work is based on the optimization of a SNEDDS platform for the topical ophthalmic delivery of RSV and MEL. To the best of our knowledge, there is no study published on MEL-loaded SNEDDS (MEL-SNEDDS) and only a few publications about RSV-loaded SNEDDS (RSV-SNEDDS), but none of them are related to the ocular field; therefore, this work can be considered a novelty in the area of nanotechnological application to therapy.

RSV- and MEL-SNEDDS were optimized using a Quality-by-Design (QbD) approach, first for the identification of the nanoemulsion zone and then for the optimization of the final formulation. Four different formulations were studied based on the type of surfactant: Tween^®^ 80, Tween^®^ 20, Cremophor^®^ EL, and Solutol^®^ HS15, respectively. Once the blank systems in terms of physico-chemical properties were optimized, they were loaded with RSV and MEL, respectively, resulting in RSV-SNEDDS and MEL-SNEDDS. The stability of the formulations allowed the identification of the optimal system, which was determined to be the one produced using Tween^®^ 80. This formulation was subsequently selected for further characterization. The systems were always prepared by reconstituting the anhydrous pre-concentrated SNEDDS (pre-SNEDDS) mixtures with simulated tear fluid (STF, pH 7.4) to mimic the conditions post-ocular instillation. A small dilution volume, a suitable temperature, and gentle agitation were chosen to produce the nanoemulsions, which were then fully characterized from a technological point of view, focusing on the requirements for ophthalmic topical formulations. Finally, cytotoxicity on rabbit corneal cells (SIRC) was assessed following a Short-Time Exposure Test (STE) protocol.

## 2. Materials and Methods

### 2.1. Materials

Mygliol^®^ 812 from IOI Oleo GmbH (Witten, Germany), isopropylmyristate (IPM) from A.C.E.F (Fiorenzuola d’Arda, Italy), Tegin^®^ O (glyceryl oleate), Capryol^®^ PGMC (Propylene glycol mono and dicaprylate NF), and Capryol^®^ 90 (propylene glycol monocaprylate NF) donated by Gattefossé SAS (Saint-Priest, France), castor oil from Sigma (Schnelldorf, Germany) were initially tested to assess the solubility of the model drugs in different oily vehicles. For the preparation of the SNEDDS, the following surfactants were used: Tween^®^ 80 (polysorbate 80), Tween^®^ 20 (polysorbate 20), and Cremophor^®^ EL (castor oil polyoxyethylene ether) were purchased from Merck (Darmstadt, Germany); Solutol^®^ HS15 (Kolliphor^®^ HS15, polyethylene glycol (15)-hydroxystearate) was gifted by BASF (Ludwigshafen am Rhein, Germany). Transcutol^®^, (2-(2-ethoxyethoxy)ethanol) gifted by Gattefossé SAS (Saint-Priest, France) was used as the co-surfactant. MEL (purity ≥98% by HPLC) was purchased from Merck (Darmstadt, Germany); RSV [trans-3,4′,5-Trihydroxystilbene; hydroalcoholic extract from Polygonum cuspidatum, Siebold et Zucc., roots; purity 99.0% by HPLC] was produced by Giellepi SpA (Seregno, Italy) and kindly gifted by Labomar SpA (Istrana, Italy). Supplementary Table S1 resumes the physico-chemical properties of the two drugs.

### 2.2. Solubility in Different Oil

Solubility studies were performed by UV spectroscopy (UH5300 UV–visible spectrophotometer, Hitachi, Chiyoda, Japan) to evaluate the solubility of RSV and MEL in different oils (see Section 2.1). An excess amount of RSV was added to 1 mL of each oil and mixed for 24 h at room temperature (r.t.). Samples were then centrifuged at 25 °C for 1 h at 10,000 rpm. The supernatant was separated, and RSV was quantified after appropriate dilutions with methanol, using a standard calibration curve at λ = 306 nm, which was linear in the concentration range 1.527–20.36 μg/mL (R^2^ = 0.9992). The same procedure was adopted for MEL, which was quantified in the samples against a standard calibration curve at λ = 224 nm, which was linear in the range of concentrations 12.844–0.803 μg/mL (R^2^ = 0.9985).

### 2.3. Development of SNEDDSs Employing the QbD Approach

#### 2.3.1. Ternary Phase Construction: Choice of Oil

A prototype panel of 14 formulations of blank SNEDDS was prepared by mixing oil, surfactant, and co-surfactant for each 8 tested mixtures: Capryol 90/Tween 80/Transcutol P, Capryol PGMC/Tween 80/Transcutol P, Capryol 90/Tween 20/Transcutol P, Capryol PGMC/Tween 20/Transcutol P, Capryol 90/Cremophor EL/Transcutol P, Capryol PGMC/Cremophor EL/Transcutol P, Capryol 90/Solutol HS15/Transcutol P, and Capryol PGMC/Solutol HS15/Transcutol P. The weight ratio of each component varied from 10% to 80%. All mixtures (in a total amount of 1 g) were prepared by stirring until the three phases were completely homogeneous. The prepared pre-SNEDDS was mixed in a 1:10 volume ratio with freshly prepared simulated tear fluid (STF, composed of 0.68 g NaCl, 0.22 g NaHCO_3_, 0.008 g CaCl_2_⋅2H_2_O, 0.14 g KCl, and distilled deionized water to 100 mL) and the ternary phase diagram was obtained by measuring the % transmittance of the obtained mixture using a UV–Visible Spectrometer at 650 nm, using distilled water as the reference. Ternary phase diagrams were drawn using the Design of Experiment (DoE) software (Design Expert^®^ 13.0, Stat-Ease Inc., Minneapolis, MN, USA). Simplex Lattice Design was used to perform this analysis, in which the emulsions obtained were classified as SNEDDS according to a clear and transparent nanoemulsion formation. The variables and responses of the design are described in Table 1.

#### 2.3.2. Construction of the Design Space

Once the oil had been chosen and the emulsion zone was understood through the construction of ternary graphs, the formulation was optimized by assembling an experimental design using I-Optimal design (Design Expert^®^ 13.0, Stat-Ease Inc., Minneapolis, MN, USA). The numerical variables entered were oil concentration (% *w/w*) (X_1_), surfactant concentration (% *w/w*) (X_2_), and co-surfactant concentration (% *w/w*) (X_3_). For each one, a minimum and a maximum level were chosen, as reported in Table 2. The effect of the surfactant type (Tween^®^ 80, Tween^®^ 20, Cremophor^®^ EL, and Solutol^®^ HS15) as categorical variable (X_4_) was also investigated on globule size (nm) (Y_1_), time of emulsification (s) (Y_2_), and % transmittance (Y_3_) after reconstitution of SNEDDS with STF (in 1:10 volume ratio). The type of oil and co-surfactant were constant for all the experiments. A constraint was added to the design, i.e., the sum of the terms should be equal to 100. Table 2 summarizes the factor and their levels and the responses used to build the experimental domain.

#### 2.3.3. Characterization of SNEDDS (Particle Size, Time of Emulsification and % Transmittance)

The preconcentrate formulation was diluted with STF (pH 7.4) at a 1:10 volume ratio under soft stirring at 30 rpm and at 35 °C to resemble the corneal surface conditions and eyelid blinking. The formed nanoemulsion was checked for mean particle size (Z-ave), time of emulsification, and % transmittance as responses of the DoE. Further characterization parameters are reported below. 

Z-Ave was measured by Photon correlation spectroscopy (PCS, Zetasizer Nano S90; Malvern Instruments, Malvern, UK) at a 90° angle of detection, at 25 °C with a 4 mW He-Ne laser operating at 633 nm. All measurements were performed in triplicate, and the results were expressed as mean ± standard deviation (SD). 

Time of self-emulsification was monitored by adding SNEDDDS to STF until the formation of a clear, transparent, blueish-tinted nanoemulsion was visually appreciable. The time for transparent blueish tint appearance was registered with a chronometer and noted. 

The formed nanoemulsions were also checked for % transmittance to determine the optical clarity using a–Visible Spectrometer set at 650 nm, using distilled water as the reference. Each measurement was made in triplicate.

#### 2.3.4. Optimization of SNEDDS

SNEDDS optimization was performed using the “desirability tool” provided by the Design-Expert^®^ 13.0 software(Stat-Ease Inc., Minneapolis, MN, USA). The desirability parameter was considered for three responses included in the experimental design: Z-Ave, time of emulsification, and % transmittance. The desirability values ranged from 0 (undesirable) to 1 (desirable). The levels of all independent variables were then automatically combined to identify the conditions within the experimental optimal domain. Four blank formulations were optimized for each surfactant (A = Tween^®^ 80; B = Tween^®^ 20; C = Cremophor^®^ EL; D = Solutol^®^ HS15).

### 2.4. Preparation of RSV-SNEDDS and MEL-SNEDDS

RSV-SNEDDS (AR, BR, CR, DR, where A, B, C, D indicate the different surfactants and R indicates the addition of RSV) were prepared by mixing the oil (Capryol^®^ PGMC), respective surfactant (A = Tween^®^ 80; B = Tween^®^ 20; C = Cremophor^®^ EL, and D = Solutol^®^ HS15), and cosurfactant (Transcutol^®^ P) at predetermined amounts, defined by optimization, until reaching a transparent and homogeneous solution. An appropriate amount of RSV (2 mg/g) was then added, and the blend was mixed at room temperature using a magnetic stirrer until complete dissolution of RSV. The prepared formulations were stored for further studies out of the light. The same procedure was used to prepare MEL-SNEDDS in order to achieve four different formulations (AM, BM, CM, DM) with 2 mg/g of MEL in the mixtures.

### 2.5. Characterization of Optimized SNEDDS

SNEDDS were reconstituted with a ten-fold volume of STF under faint stirring (about 30 rpm) prior to characterization to simulate the blinking phenomenon and mimic their behavior in the ocular environment after administration. The Z-ave, polydispersity index (PDI), and zeta potential (ZP) values of SNEDDS after reconstitution were measured by PCS analysis, as previously described (Section 2.3.3). Analogously, the time of self-emulsification and optical clarity (% transmittance) were measured as described for the non-optimized systems (Section 2.3.3).

### 2.6. Stability Evaluation 

Stability studies were performed following the ICH Q1A (R2) guidelines (Stability testing of new drug substances and products) [20]. All samples (SNEDDS, RSV-SNEDDS, and MEL-SNEDDS) were evaluated after storage at different conditions (4 °C, 25 ± 2 °C/60 ± 5% R.H, and 40 ± 2 °C/75% ± 5% R.H.) in a climate chamber (Blinder GmbH, Tuttlingen, Germany). Z-ave, PDI, and ZP were measured every month and up to 3 months, as previously described.

### 2.7. Stability in Ocular Environment

To evaluate the stability of SNEDDS in the ocular environment, the pre-SNEDDS were diluted in a ratio of 1 to 10 with STF and placed in a climatic chamber at 37 °C. At time intervals (5, 10, 15, 20, 30, 60, 120, and 180 min), they were analyzed for size, PDI, and % transmittance.

### 2.8. pH, Osmolarity, and Viscosity Determination

The pH of SNEDDS formulations was measured at 25 °C by a Mettler Toledo pH-meter (Columbus, OH, USA). The instrument was calibrated using standard Mettler Toledo buffer solutions (pH 4.01 ± 0.02; 7.00 ± 0.02, and 10.00 ± 0.02; slope 99.8%). Each measurement was performed in triplicate. The osmolality (mOsm/Kg) of the samples was determined using a cryoscopic osmometer (Osmomat, mod. 030-D, Gonotec, Berlin, Germany). Deionized water (consistent with the 0 mOsmol point) and a 300 mOsmol/L calibration standard (consistent with the 300 mOsmol point) were used for a 2-point calibration. Each sample was analyzed in triplicate.

Dynamic viscosity of SNEDDS preconcentrates was obtained by rheological measurements with a rheometer (Haake Mars, ThermoFisher Scientific, Darmstadt, Germany). A C35/1° Ti-plate measuring system was used. The measurement parameters were as follows: shear stress 1–50 Pa, frequency 1 Hz, temperature 25 ± 0.5 °C.

### 2.9. FT-IR Analysis 

FT-IR spectrophotometer (Perkin-Elmer Spectrum RX I, Waltham, MA, USA) was employed for the measurement of pure materials (Capryol^®^ PGMC, Transcutol^®^ P, Tween^®^ 80, RSV, and MEL), blank SNEDDS, RSV-SNEDDS, and MEL-SNEDDS. The tool was equipped with an attenuated total reflectance (ATR) accessory, a diamond window, and zinc selenide crystal (diamond/ZnSe). For each sample, 64 scans were collected at room temperature over the 4000–600 cm^−1^ range at a resolution of 4 cm^−1^. Any background absorption was subtracted before each analysis.

### 2.10. Mucoadhesion Study 

The mucoadhesion study was performed with the formulation produced with Tween^®^ 80 (A). Pre-SNEDDS were reconstituted with STF (1:10 by volume), and the formed nanoemulsion was incubated with porcine mucin (0.1%, *w/v*) dispersion (1:1, *v/v*) in STF at 37 °C. Mean globule size, % transmittance, and ZP were measured after 0, 30 min, 1, 2, and 24 h of incubation.

### 2.11. Drug Entrapment Efficiency (EE%)

RSV-SNEDDS and MEL-SNEDDS (1 mL) with a concentration of each drug of 2 mg/g were centrifuged for 30 min at r.t and at 10,000 rpm. The supernatant was suitably diluted with methanol and analyzed by UV spectrophotometry. The drugs were quantified at a wavelength of 306 nm for RSV and 224 nm for MEL. EE% was calculated as follows for each sample:

EE% = (total μg drug − μg of drug in the supernatant)/(total μg drug) × 100

### 2.12. Cloud Point Measurements

The cloud point typically refers to the temperature at which a mixture of surfactants undergoes a phase transition, leading to the formation of a cloudy or turbid appearance due to the separation of the emulsion components. This corresponds to the breaking of the emulsion, and it is visually noticeable because the nanoemulsion becomes turbid. The analysis was carried out as follows: different dilutions of SNEDDS/STF were prepared (1:10, 1:50, 1:100, 1:200, 1:300, *v/v*). Each formulation was then placed in a thermostat bath, and the temperature gradually raised. When the formulation became turbid, the temperature was recorded, and the sample was subjected to turbidimetric UV analysis at 650 nm to confirm the change in appearance [21]. The breaking of the nanoemulsion was corroborated by the measurement of Z-ave and PDI changes. 

### 2.13. High-Performance Liquid Chromatography (HPLC) Method for the Quantification of MEL

HPLC analysis was performed using an Agilent 1100 binary pump (Agilent Technologies Inc., Wilmington, DE, USA), a 1100 micro vacuum degasser, a HP 1050 Autosampler, and a HP 1050 variable wavelength detector (operated at 235 nm). The chromatographic separation was achieved on a Supelco Supelcosil^TM^ LC-SI analytical column (4.6 mm × 250 mm, 5 μm) (Supelco Inc., Bellefonte, PA, USA) by an isocratic elution of a formic acid 0.1% (*v/v*) solution in Milli-Q^®^ water and a formic acid methanol solution (0.1%, *v/v*) (40:60, *v/v*). Effluent was monitored at a wavelength of 278.4 nm, with a flow rate of 1 mL/min; the injection volume was 5 µL, retention time of MEL was 3.5 min. The column was maintained at 45.0 ± 0.2 °C throughout the whole analysis.

The standard calibration curves were prepared at different dilutions of MEL in Milli-Q^®^ water/methanol (1:1, *v/v*). The linear regression coefficient determined in the range 0.5–200 μg/mL was 0.9999. 

### 2.14. High-Performance Liquid Chromatography (HPLC) Method for the Quantification of RSV 

HPLC analysis was performed using an Agilent 1100 binary pump (Agilent Technologies Inc., Wilmington, DE, USA), a 1100 micro vacuum degasser, a HP 1050 Autosampler, and a HP 1050 variable wavelength detector (operated at 235 nm). The chromatographic separations were achieved on a ZORBAX^®^ Eclipse XDB-C18 (2.1 mm × 100 mm, 1.8 μm) (Agilent, USA) by using isocratic elution of Milli-Q^®^ water and acetonitrile (75:25, *v/v*). Effluent was monitored at a wavelength of 310 nm, with a flow rate of 0.3 mL/min; the injection volume was 5 µL; retention time of RSV was 5.2 min. The column was maintained at 45 °C throughout the analysis.

RSV standard calibration curves were prepared in Milli-Q^®^ water/EtOH (20% *v/v*) with a linear regression coefficient determined in the range 0.1–100 μg/mL was 0.9989. All procedures were carried out to protect the sample from light.

### 2.15. In Vitro Release Test 

The in vitro release profile of MEL-SNEDDS and RSV-SNEDDS in STF, pH 7.4, was performed through a dialysis bag method. One milliliter samples were transferred into a dialysis tube (Spectrum™ Spectra/Por™ membranes, MWCO 3.5 kDa; Fisher Scientific Italia, Segrate, Milan, Italy). The bag was incubated in 4 mL of medium and maintained under magnetic stirring at 37 °C for up to 8 h. At predetermined time points, 2 mL of the release solution was withdrawn and replaced with the same volume of fresh medium. The taken specimens were immediately put into liquid nitrogen and freeze-dried (BuchiLyovaporTM L-200 Freeze Dryer, Fisher Scientific Italia, Segrate, Milan, Italy) for 24 h at 0.1 mbar. 

In order to extract MEL, each dried sample was dissolved in 0.5 mL of Milli-Q^®^ water/methanol (1:1, *v/v*) by vortexing for 5 min. The sample was then centrifuged at 10,000× *g* at 4 °C for 30 min to remove the lipid matrix residue, and the supernatant was collected and injected into the HPLC to measure the MEL content. The experiment and HPLC analyses were performed in duplicate. The concentration of MEL was quantified as reported in Section 2.11.

To extract RSV, each dried sample was dissolved in 0.3 mL of Milli-Q^®^ water/ethanol (1:5, *v/v*) by vortexing for 5 min. The sample was then centrifuged at 10,000× *g* at 4 °C for 30 min to remove the SNEDDS matrix residue, and the supernatant was collected and injected into the HPLC to measure the RSV content. The whole procedure was carried out, protecting the sample from light. The experiments and the HPLC analyses were performed in duplicate. The concentration of RSV was quantified as reported in Section 2.12.

The in vitro release data of SNEDDS were analyzed according to various kinetic models:

Zero-order model: R = Kot
First-order model: R = 1 − e − kt
Higuchi model: R = KH t1/2
Hixson–Crowell model: Wo1/3 − Wt1/3 = KHCt
Korsmeyer–Peppas model: R = kKP tn

The amount of the MEL and RSV released at time t; ko, k, kH, KHC, and kKp (k are the rate constants for the different above models) was expressed by R. Wo is the initial amount of MEL/RSV, and Wt is the amount of drug at time t; n is the release exponent in the Korsmeyer–Peppas model [22]. The model with the highest correlation coefficient (R2) was selected to describe the mechanism of MEL and RSV release. Calculations were made on the linear part of the release curves (from 0.5 h forward).

### 2.16. Cell Cultures and Viability Assay (MTT)

The test was performed following the short-time exposure test (STE) [23,24]. The Statens Serum Institut Rabbit Cornea (SIRC) epithelial cells (ATCC CCL-60) were grown in specific medium, Eagle’s Minimum Essential Medium (ATCC 30-2003TM), complemented with 10% fetal bovine serum (FBS) and 1% penicillin–streptomycin (P/S) and maintained at 37 °C and 5% CO_2_, as previously described [25]. Fresh medium was replaced every day, and when the confluence was reached, the cells were seeded into 96-well plates at a density of 1 × 10^4^ cells/well in 100 μL of medium for 24 h. Subsequently, the cells were treated with different concentrations of SNEDDS, RSV-SNEDDS and MEL-SNEDDS in a medium supplemented with 1% FBS for 5 min. Then, the viability assay was performed by adding 100 μL of 3-[4,5-dimethylthiazol-2-yl]-2,5-diphenyltetrazolium bromide (MTT) (ACROS Organics, Antwerp, Belgium) solution for 3 h at 37 °C and 5% CO_2_, as previously described [26]. At the end of incubation, the supernatant was removed and replaced with 100 μL of DMSO in order to dissolve the formazan salts produced by mitochondria. The amount of formazan formed by the cleavage of the yellow tetrazolium salt MTT, proportional to the number of viable cells, was measured by using a microplate reader (Biotek Synergy-HT, Winooski, VT, USA) at 550–600 nm. Six replicate wells were used for each group, and at least three separate experiments were performed.

### 2.17. Statistical Analysis 

Characterization data are representative of three separate experiments, and statistical analysis was carried out by two-way ANOVA followed by Dunnett’s test. For the mucoadhesion study, statistical analysis was performed by Graphpad Prism 9.5.0 (GraphPad Software, Inc., San Diego, CA, USA) through two-way ANOVA and Šídák’s multiple comparisons tests. For the MTT assay, results are representative of at least three independent experiments, and values are expressed as a percentage of control (** *p* < 0.01 or *** *p* < 0.001 vs. STF, as determined by one-way ANOVA followed by Tukey–Kramer post hoc test).

## 3. Results and Discussion

### 3.1. Solubility of Drugs in Various Oils

**Figure 1:**
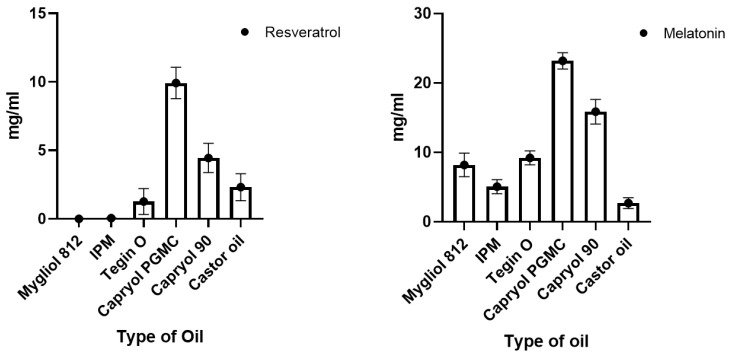
Solubility of RSV and MEL in different oils.

The solubility of RSV and MEL was determined to evaluate which oil better solubilized the active compounds. The set of tested oils was selected based on those most commonly used in the literature for the preparation of SNEDDS (Figure 1). Among the six assayed oils, Capryol^®^ PGMC showed the highest solubility (9.92 ± 1.30 mg/mL for RSV and 23.18 ± 0.88 mg/mL for MEL). Capryol^®^ PGMS was followed by Capryol^®^ 90 with the capacity to solubilize 4.47 ± 1.07 mg/mL of RSV and 15.86 ± 1.70 of MEL. The choice of the oily vehicle is a crucial step; indeed, the formulation must consist of components that solubilize the drug and form a monophasic mixture when shaken together. Furthermore, Capryol^®^ 90 and, even more, PGMC possess the ability to spontaneously form clear emulsions when mixed with surfactants with high HLB, such as polysorbates (Tween^®^). Both have been extensively investigated for the development of nanoemulsion and SNEDDS, as well as microemulsions and SMEDDS [27,28,29,30]. The emulsion formed must be clear and limpid and must not allow the drug to precipitate [31]. Therefore, the choice of the oil in which the drug is most soluble is highly relevant. These two oils were chosen to continue the study with the search for the emulsion zone using the ternary graph. 

### 3.2. Ternary Plot Diagram Construction

**Figure 2:**
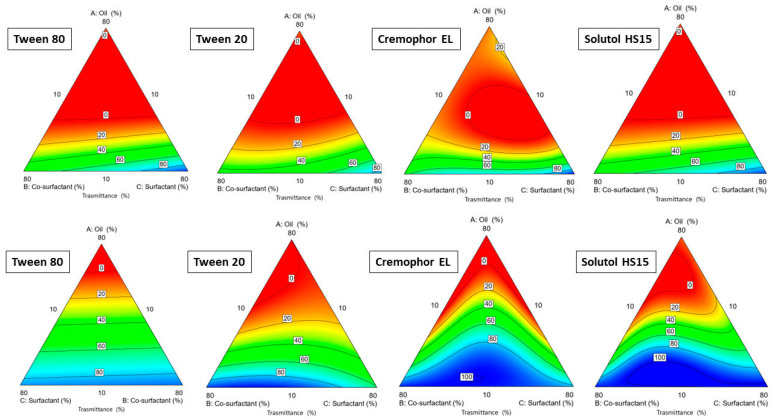
Ternary plot diagrams obtained by the mixtures of Transcutol^®^ P, different surfactants and Capryol^®^ 90 (upper row) and Capryol^®^ PGMC (bottom row).

The ratio between the phases varied in each experiment, keeping the sum of the three phases constant at 1 g in the final SNEDDS. The % transmittance was evaluated in a range from 0.1 to 100% for the various emulsions produced. The blue zone indicated an emulsion with a high % transmittance, followed by the green, orange, and finally red zones, which indicated increasingly lower transmittances down to 0.1 (red zone). Obtaining a transmittance close to 100% suggested a globule size below 100 nm. As Figure 2 shows, Capryol^®^ PGMC allowed to produce emulsions with a high degree of transmittance. When comparing the graphs, it is possible to see a higher concentration of blue areas in the graphs produced with Capryol^®^ PGMC. Both are propylene glycol mono- and diesters of caprylic acid (C8) but with different fractions of monoesters [32]. This slightly different composition, combined with a different behavior with the various surfactants, gave rise to differences in terms of spontaneous nanoemulsification in favor of Capryol^®^ PGMC. It had already demonstrated its superiority over Capryol^®^ 90 in terms of self-nanoemulsification efficiency in many mixtures with different surfactants [33].

### 3.3. Construction of the Experimental Design 

The blank SNEDDS were first optimized with the experimental design. Forty-two runs were formulated to obtain all possible combinations with the four surfactants. For the construction of the experimental design, the following numerical variables were chosen: oil concentration (% *w/w*), surfactant concentration (% *w/w*), and co-surfactant concentration (% *w/w*). This is a crucial pre-formulation point. Choosing in which ratios the three phases should co-exist allows the final goal to be achieved: a homogenous formulation that emulsifies in a short time without giving the presence of drug precipitate. Finally, the type of surfactant was chosen as the categorical variable. Four surfactants with a common characteristic, i.e., hydrophilicity (HLB > 12), were tested, and they were included in the design as they favor the occurrence of O/A emulsions. Tween^®^ 80 (HLB = 15), Tween^®^ 20 (HLB = 16.7), Cremophor^®^-EL (HLB = 13), and Solutol^®^ HS15 (HLB = 15) are non-ionic surfactants, and they are considered less toxic and more cytocompatible than ionic surfactants [34]. They also had a very good ability to emulsify Capryol^®^ PGMC. In fact, the possibility of quick emulsification in contact with small volumes of an aqueous fluid, such as in the ocular surface, is possible using a surfactant with a high HLB. The choice of testing four surfactants was linked to the consideration that the emulsification ability of each surfactant is typically influenced not only by the HLB and structure but also by their physical behavior once mixed with the other two components [35].

#### 3.3.1. Effect of Independent Variables on Globule Size 

DoE involves creating a structured plan for understanding the relationship between the independent variables (factors) and dependent variables (responses) in a system. Once all the experiments suggested by the software are completed, it provides polynomial equations, one for each response, to determine the influence of each factor in the response and the interaction between factors and responses. The equation below represents the influence of each factor on the size of SNEDDS (1). This was obtained once all the formulations of SNEDDS were prepared and analyzed for particle size. The obtained globule size for all experiments was in the range 13.29 ± 0.135–455.1 ± 15.36 nm.

Size (Y_1_) = 152.98044384037 + 111.97043203167 X_1_ + 84.068897927114 X_3_ − 5.413763606443 X4 [1] + 21.113050603648 X4 [2] − 48.03416483216 X1×4 [1] + 8.8903231966525 X1X4 [2] + 45.844330806972 X12 (1)

**Figure 3:**
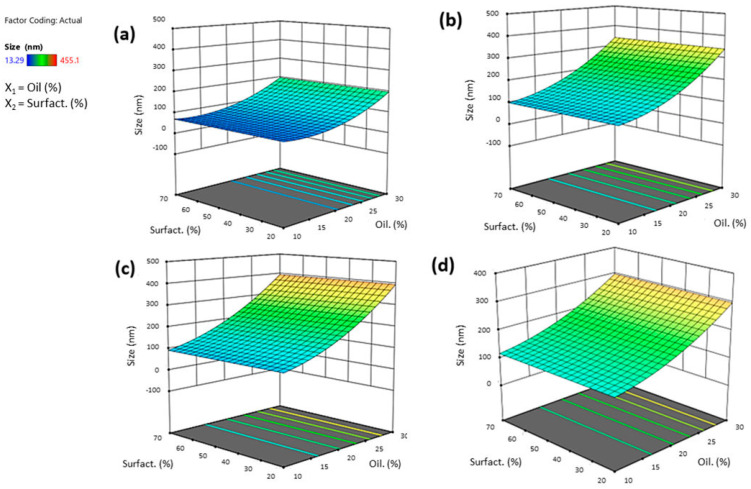
Three-dimensional surface of the effect of independent variables (surfactant vs. oil concentration) on the globule size of SNEDDS after reconstitution with STF (1:10 by volume) using Tween^®^ 80 (**a**), Tween^®^ 20 (**b**), Cremophor^®^ EL (**c**), or Solutol^®^ HS15 (**d**).

The ANOVA analysis of the full regression models showed that only some factors were statistically significant; thus, statistically non-significant terms were removed except for terms needed for hierarchy in the reduced regression model. The quality of the fit of the experimental data using the reduced quadratic models was assessed based on several statistical criteria. Taking into account the R^2^ parameter, the quadratic model was considered the most significant since Adjusted R^2^, given by the software, was close to Predicted R^2^. Moreover, the generated model F-value of 9.77 implies the model is significant. The term B, which refers to co-surfactant concentration, was excluded by the model because it had no significant influence on globule size. When both oil and surfactant concentrations were increased, globule size decreased due to the oil’s dominant influence over the surfactant. Indeed, oil concentration appeared to be the factor most influential on the mean size response, with an estimated coefficient equal to +111.97. A positive coefficient revealed a direct relationship between the variable and the response. As shown by the response surface graphs, an increase in oil concentration produced larger particles (Figure 3). Smaller particle sizes were observed when using Tween^®^ 80, as represented in response surface graphs (Figure 3). Tween 80^®^ allows for better stabilization of nanoemulsions, forming a protective layer around the micelles and preventing their aggregation. It also has a controlling role in the whole emulsification process, reducing the surface energy and leading to the inhibition of crystal growth during the entire process [36]. The square bracket in polynomial Equation (1) indicates an optional third parameter associated with the level of that factor implied in the polynomial regression. For example, the influence associated with the type of surfactant depends mainly on the first level with Tween^®^ 80 and on the second level and thus on Tween^®^ 20 (a result that perfectly corroborates the response surface plots below).

#### 3.3.2. Effect of Independent Variables on Time of Emulsification 

Once the values are inserted in the software, it generates a polynomial Equation (2) that relates the influence of each factor with the time of emulsification derived from all SNEDDS prepared. The obtained time of emulsification for all experiments was in the range of 31 ± 1.2–134 ± 5.50 s. As demonstrated in the polynomial equation, the surfactant concentration and the interaction between surfactant type and its concentration were identified as the most influential factors regarding the time of emulsification response.

Time of emulsification = 75.162927762315 + 0.12557087841826 X_1_ + 23.558956914706 X_2_ + 12.877171801241 X4 [1] + 5.5936332662947 X4 [2] + 0.43202022289143 X1X4 [1] + −8.844328114505 X1X4 [2] + 20.231882039157 X2X4 [1] + 13.526675893769 X2X4 [2] (2)

As suggested by the software, the reduced 2FI model was considered significant since Adjusted R^2^ was close to Predicted R^2^. The generated F-value of 9.30 for this model implies the model is significant. Some factors were deleted by the model since they were considered not significant. One of them was the co-surfactant concentration, whose influence was considered not significant in changing the time of emulsification parameter.

**Figure 4:**
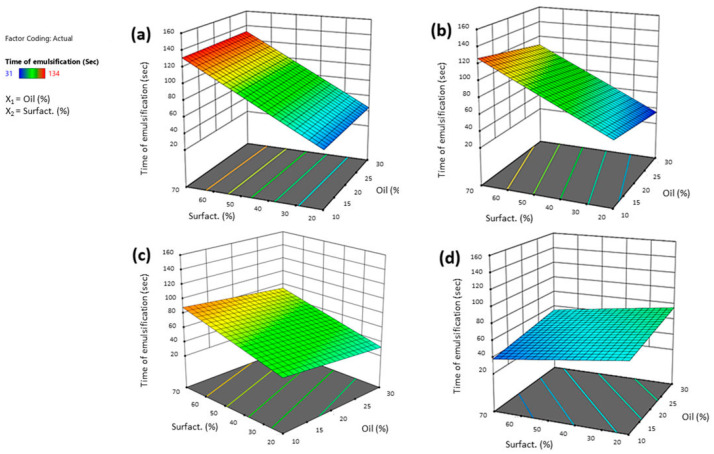
Three-dimensional surface of the effect of independent variables (surfactant vs. oil concentration) on the time of emulsification of SNEDDS after reconstitution with STF (1:10 by volume) using Tween^®^ 80 (**a**), Tween^®^ 20 (**b**), Cremophor^®^ EL (**c**), or Solutol^®^ HS15 (**d**).

Factors X_2_ had a positive influence on this response (+25.66). An increasing amount of surfactant increased the emulsion time. The right surfactant concentration allows accessibility to the oil/water interface, reducing interfacial tension and thus the possibility of obtaining an emulsion in a short time. But when the surfactant is excessive, the formulation becomes viscous, and the moment fluid comes into contact with the SNEDDS, the emulsion time of the latter increases. It is worth noting from the graphs that the interaction between surfactant type and oil is important (Figure 4). In the case of both Tween^®^ 20 and 80, the emulsion time increased with increasing surfactant concentration, without any influence from the oil concentration. In the case of Cremophor^®^ and Solutol^®^ HS 15, instead, the increase in oil concentration must also be taken into account: the emulsion time increased when the surfactant amount decreased, and the oil concentration increased at the same time. The formation of a mixture that was more viscous (such as with Tween^®^ 80 and Tween^®^ 20) could slow down the movement of the liquid between the phases and result in the formation of droplets for a longer time. Higher viscosities tend to slow down the emulsification rate, as reported by Nasr et al. [37].

#### 3.3.3. Effect of Independent Variables on Transmittance% 

The obtained % transmittance for all experiments was in the range 0.1–100 ± 0.10%. Just as in the case of size, for transmittance, the most influential factor was the amount of oil. Transmittance and particle size are closely linked: a high optical clarity (90–100%) stands for globules with a size smaller than 50 nm. In contrast, a low transmittance indicates that the emulsion formed is not clear and will certainly contain particles of the order of more than 100 nm. 

As for other responses, the equation below (3) represents the quantitative effect of the process variables (oil, surfactant, and co-surfactant) and their interaction on the % transmittance. In this case, all the factors investigated exerted a significant influence on the response. The linear model (F-value of 20.94) was considered significant, with Adjusted R2 being close to Predicted R2 and, as the equation below shows, the most influential factor was oil concentration.

Transmittance = 38.873268681425 − 36.562914111227 X_1_ + 11.233423582655 X_2_ − 26.931299436827 X_3_ + 26.002042325709 X4 [1] − 12.538357360177 X4 [2] (3)

**Figure 5:**
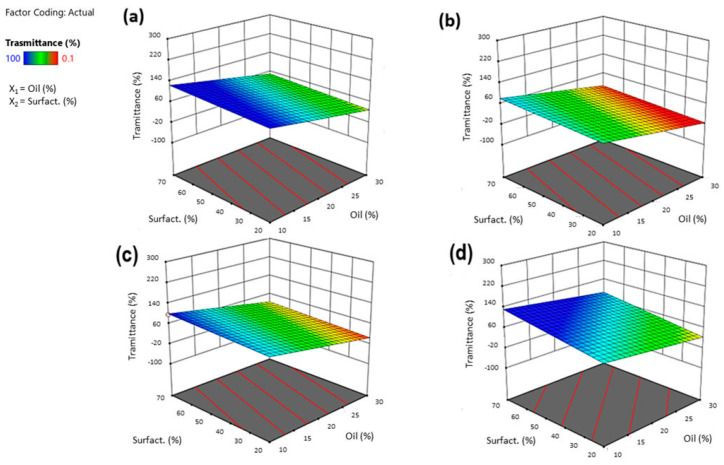
Three-dimensional surface of the effect of independent variables (surfactant vs. oil concentration) on the % transmittance of SNEDDS after reconstitution with STF (1:10 by volume) using Tween^®^ 80 (**a**), Tween^®^ 20 (**b**), Cremophor^®^ EL (**c**), or Solutol^®^ HS15 (**d**).

Given the negative estimated coefficient (−36.56) of X_1_, the relationship between % transmittance and oil was indirect: higher oil concentrations produced milky nanoemulsions with low transmittance (~0.1%), which, in turn, was synonymous with the presence of larger globules (Figure 5). The coefficient of the co-surfactant was also negative, meaning that with increasing concentrations of the co-surfactant, the transmittance value decreased. Formulations with a high percentage of Transcutol^®^ P gave emulsions with a milky appearance, with transmittance values less than 30%. This could be attributed to the low HLB of Transcutol^®^ P, around 4–5, that allowed rapid emulsification in combination with Tween^®^ 80 but produced milky emulsions. Indeed, Transcutol^®^ P is sometimes used as an oil phase for the development of SNEDDS; and, as discussed above, an increase in the oil phase generated a higher transmittance.

### 3.4. Optimization Phase 

For the optimization of SNEDDS, different criteria were set to suit the ocular administration by topical instillation. Therefore, it was decided to (i) minimize the oil concentration, as it affected both size and transmittance; (ii) increase the surfactant concentration, as this choice produced smaller sizes; (iii) minimize the concentration of co-surfactant, given its influence on % transmittance and clarity of the nanoemulsions. 

The surfactant type remained in the range to have more options to optimize the formulations: a small size was preferable, as it would improve the diffusion towards the deeper structures of the eye globe.

Preferably, a time of less than 20 s would be perfect since SNEDDS, once applied on the ocular surface, must emulsify with the tear fluid very quickly. A long time would not allow the SNEDDS to emulsify before being drained away. Finally, high transmittance was important since this would prevent blurred vision when the formulation was instilled. The selected optimization criteria are shown in Table 3.

Once the optimization criteria were chosen, the software generated a set of optimized formulations, which were sorted according to desirability values. Desirability gives an idea of how well the predicted formulation fits with the chosen parameters. It ranges from a value of 0 to 1. Four formulations with the highest desirability were selected, one for each surfactant, in order to assess the stability of the active ingredients in each of them (Table 4).

The produced four formulations were characterized in terms of size, emulsification time, and % transmittance (Table 5). After calculating the % error between the predicted response and that obtained experimentally, an error of less than 10% was registered for all responses. The chosen model could thus be considered highly predictive for the formulation of SNEDDS [38].

### 3.5. Characterization of SNEDDS, RSV-SNEDDS, and MEL-SNEDDS 

MEL and RSV were loaded into the SNEDDS in order to achieve a therapeutic concentration of drugs after reconstitution. Literature data indicate that MEL at 10^−4^ µM attenuates oxidative stress and inflammation of Müller cells via activating the SIRT-1 pathway [39,40]. RSV at 100 µM increases SIRT-1 overexpression in retinal pigment epithelium [41,42].

**Figure 6:**
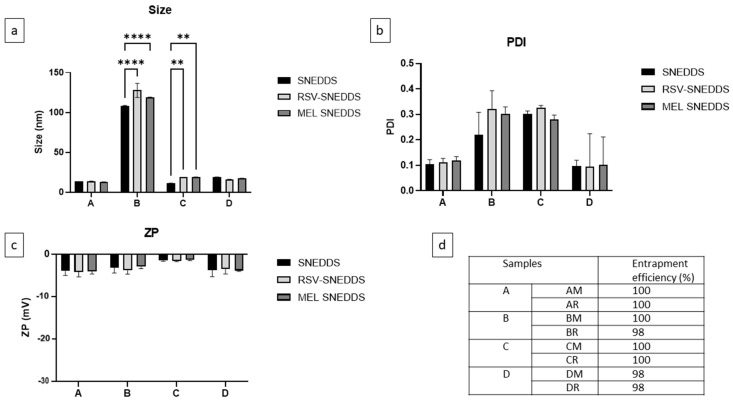
Characterization of SNEDDS in terms of (**a**) size, (**b**) PDI and (**c**) ZP before and after loading with RSV and MEL; (**d**) Entrapment efficiency (EE%) of drug-loaded samples A, B, C, and D. ** *p* < 0.01, **** *p* < 0.0001.

Technological and physico-chemical parameters remained unchanged when the formulations were loaded with drugs with respect to the blank systems (Figure 6). Furthermore, no precipitate was observed after the formation of the extemporaneous nanoemulsion. The resultant small droplet size would provide a large interfacial surface area for drug release and absorption. The results obtained in terms of size seemed optimal. The passage from the corneal surface to the vitreous humor is allowed for molecules smaller than 500 nm since the mesh size of the bovine vitreous has been estimated at ~550 nm. However, to allow diffusion without minimal steric hindrance and to obtain formulations that do not cause irritation or blurred vision upon administration, a size less than 200 nm is preferable [43]. Furthermore, the slightly negative charge of the SNEDDS obtained and their small size, especially in the case of systems A, C, and D, allowed them to move toward the retina. Studies demonstrated that small-sized (≈50 nm) PEG-coated anionic liposomes showed the most extensive cellular distribution and localization in the retina [44]. All the produced SNEDDS had a very small size, slightly higher in the case of SNEDDS formed with Tween^®^ 20. A PDI of less than 0.3 always indicated a good homogeneity of the formulation. The slightly negative ZP was attributable to the materials used; however, none of the SNEDDS displayed a net charge and were very close to neutral. 

The entrapment efficiency of SNEDDS loaded with RSV (AR, BR, CR, DR) or MEL (AM, BM, CM, DM) was high for all samples. No precipitate was observed after dilution with STF as proof that all the active ingredient was encapsulated and retained in the lipid mixtures. The pH and osmolarity values were assessed upon dilution with STF; the small dilution gave an idea of the actual SNEDDS values. All SNEDDS showed pH values between 6.9 and 7.5 and osmolarity in the range of 0.281 to 0.320 Osm/kg after reconstitution with STF (1:10 by volume). These values could be considered optimal for an ophthalmic liquid formulation that should be non-irritating once applied [45]. Higher or lower values of osmolarity could cause irritation and extensive lachrymation, provoking a rapid wash-out of the solution and consequently a poor drug local bioavailability [46].

The values of viscosity obtained for formulations A, AR, and AM were in the range of 15–58 mPa as a function of time. A viscosity value up to 50 mPa-s is generally considered tolerated for an ocular formulation. Thus, the tested SNEDDS appears to be suitable for ocular administration in terms of viscosity without presumption of impairment of normal visual function. 

### 3.6. Stability in Simulated Ocular Environment 

**Figure 7:**
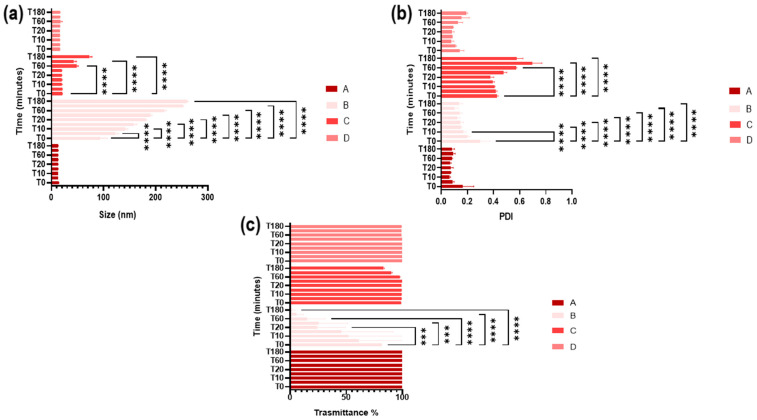
Stability in ocular environment of formulations A, B, C and D in terms of (**a**) size, (**b**) PDI and (**c**) % transmittance (*** *p* < 0.001 or **** *p* < 0.0001 vs. T0, as determined by two-way ANOVA followed by Dunnett’s multiple comparisons test).

Characterization of SNEDDS in the potential ocular environment can be useful for evaluating their potential behavior in vitro and in vivo. The temperature, the pH, and the ions present in the tear fluid could influence the interaction of the systems with the cellular compartment [47]. Therefore, the colloidal stability of the SNEDDS placed in contact with a potential ocular environment (STF, 37 °C in a climatic chamber) was evaluated for a maximum time of 180 min. At specific various time intervals, they were analyzed in terms of Z-ave, PDI, and % transmittance. Significant changes would indicate an instability of the systems. Figure 7 highlighted high stability for all formulations except for C and B. At 37 °C, the latter formulation exhibited an increase in size and % transmittance over time, turning milky already after 5–10 min. The increase in size observed in both formulations may be attributed to an aggregation phenomenon, suggesting minimal stability within three hours. Formulations A and D exhibit considerable stability throughout the analysis period. The absence of particle aggregation is crucial as this phenomenon could potentially induce irritative effects, leading to rapid drainage of the systems before interacting with the ocular surface, thereby compromising the effectiveness of the system.

### 3.7. SNEDDS Stability Evaluation

The stability study of the SNEDDS was carried out according to the ICH guidelines for the stability testing of new drug substances and products [20] under different storage conditions. Like all new drug products, nanomedicines should demonstrate constancy of physico-chemical and microbiological parameters under suitable thermal and humidity storage conditions and durations prior to their registration [48]. When nanomedicines do not contain or are not decorated with biotechnological molecules but incorporate only a drug and/or an imaging agent, as in the present work, stability testing can follow the indications of the ICH guidelines Q1A (R2) and Q1C (in the case of new dosage forms of already registered products) [48].

**Figure 8:**
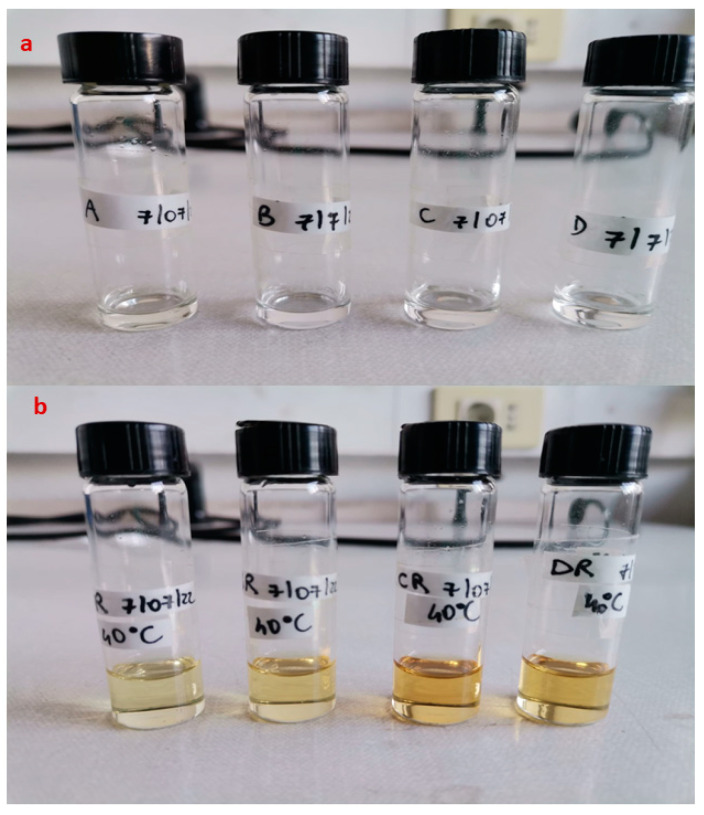
(**a**) Blank SNEDDS after 6 months after storage at 40 °C and (**b**) RSV-SNEDDS after one week of storage at the same temperature.

Drug-loaded SNEDDS were, therefore, placed at three different temperatures: 4 °C, 25 °C (at 60% R.H.), and 40 °C (at 75% R.H.). SNEDDS loaded with MEL showed no sign of visual instability; RSV-SNEDDS instead showed a strong degradation of RSV already after one week at 25 °C, but especially at 40 °C, as suggested by the development of a yellow color in the vials (Figure 8b). The instability could be definitely associated with the encapsulated RSV since, as Figure 8a shows, the corresponding blank SNEDDS did not show the same alterations until 3 months of storage.

**Figure 9:**
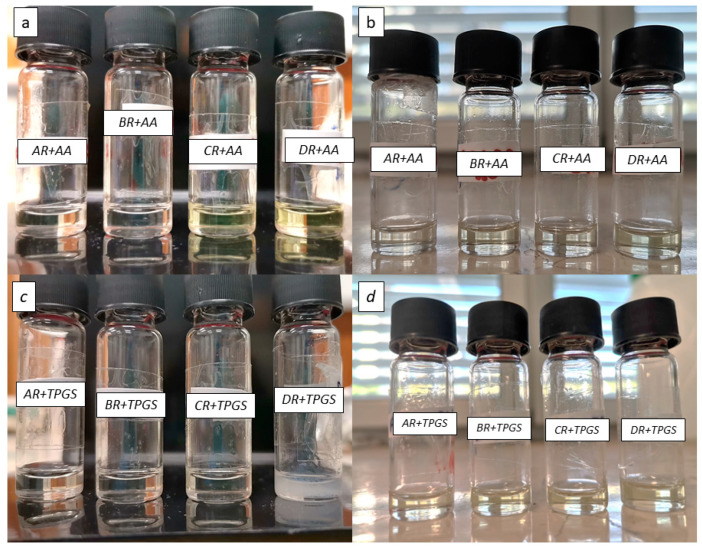
RSV-SNEDDS after 3 months of storage (**a**) with ascorbic acid at 25 °C and (**b**) at 40 °C, or (**c**) with TPGS at 25 °C and (**d**) at 40 °C.

Blank SNEDDS can be defined as stable systems since formulations that do not show significant changes at 40 °C for at least 6 months (accelerated stability conditions) can be defined as stable for a hypothetical time frame of at least one year [20]. Literature studies confirm the stability of RSV at temperatures between 4 °C and −20 °C and also confirm the high possibility of degradation at temperatures above 25 °C [49,50]. High temperature favored oxidation, epimerization, hydrolysis, and/or polymerization of stilbenes [51]. The increase in temperature induces a change in the appearance of the formulation with a typical color change that is evident as the temperature rises (40 °C > 25 °C). Indeed, oxidation of RSV at high temperatures produces a change in the molecule that leads to a light yellow to dark yellow color. This degradation was also influenced by the materials used, as shown in Figure 8b [52]. The color change produced during storage at high temperatures is due to the degradation of RSV. The degradation of RSV produces aromatic degradation under products that absorb at wavelengths above 300 nm that can be monitored with UV-visible and are potentially responsible for the color change in the formulation. This is a phenomenon that is much more noticeable when exposing the RSV to light but is still visible when the RSV is exposed to high temperatures [53,54]. To reduce RSV alteration, the formulations were supplemented with an antioxidant agent, namely vitamin E TPGS or ascorbic acid, and their stability was monitored at 25 and 40 °C. As shown in Figure 9, the use of antioxidants increased the shelf life of the RSV-SNEDDS during a 3-month period.

**Figure 10:**
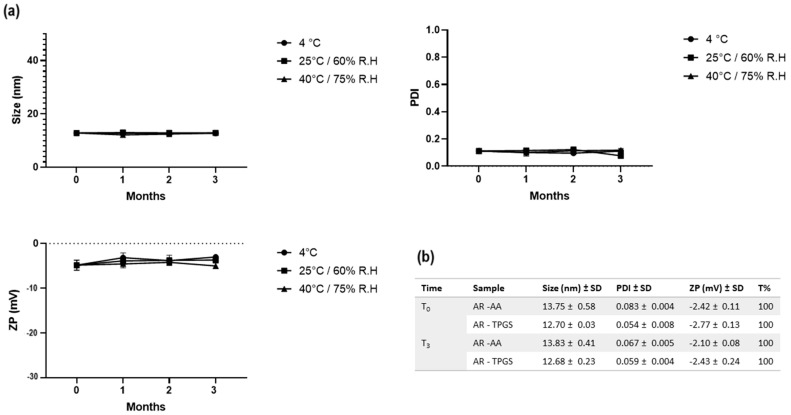
(**a**) Stability of SNEDDS at different storage conditions in terms of size, PDI and ZP. (**b**) Stability after 3 months of AR added with TPGS or ascorbic acid (AA) at concentration of 0.015% *w/w*.

As revealed by Figure 9, formulation A was much more physically stable with the addition of both antioxidants under the conditions tested. Tween^®^ 80, as a surfactant combined with an antioxidant compound, was able to prevent the degradation of RSV, as also reported by Das et al. [52]. Therefore, this formulation was chosen for the subsequent studies. In addition, stability studies showed that formulations C and D underwent gelation at the temperature of 4 °C, with increased size and PDI. Formulation B, despite being moderately stable, is the formulation that gives the largest size with increasing transmittance over time. Its instability is not suitable for ocular administration. The unloaded formulation A was subjected to stability studies under the same storage conditions. As shown in Figure 10, it was stable for up to 3 months, showing no signs of destabilization in terms of size, PDI, and ZP. The unchanging parameters for a long period highlight the high robustness of formulation A, even under accelerated conditions. The AR formulation (RSV-loaded A formulation) supplemented with an antioxidant was evaluated in terms of size, ZP, PDI, and % transmittance (cf. Table inside Figure 10), giving excellent preservation results to corroborate the visual results presented above (Figure 9). MEL-SNEDDS gave the same results also for formulations B, C, and D. Formulation A, loaded with MEL (AM), was the most stable for up to 3 months at 4 °C without antioxidant addition and at 25 and 40 °C with the addition of a small percentage (0.015% *w/w*) of TPGS or ascorbic acid. It did not show significant destabilization phenomena or signs of MEL precipitation. The size, PDI, and ZP remained unchanged for the whole tested period.

#### Measurement of Cloud Point

The cloud point represents the temperature at which an emulsion becomes cloudy and breaks down. It is important to assess whether a nanoemulsion maintains its physical stability at the body site of administration. For ocular application, the emulsion must remain stable and not break down at 35 °C, the temperature of the ocular surface. At temperatures above the cloud point, an irreversible phase separation occurs due to dehydration of the formulation. This can inhibit the efficiency of the system, induce expulsion of the drug from the formed micelles, with its precipitation, and thus affect the drug absorption and efficacy overall.

Table 6 shows that the emulsion formed after reconstitution of SNEDDS A with different ratios of STF had a cloud point above 40 °C at all the dilutions tested. This was corroborated by the size and PDI value, measured just after the dilution and gathered in the same table. The results demonstrated excellent temperature stability up to 47 °C in the case of a small dilution, as might occur on the ocular surface, and even greater stability at higher dilutions, which might be important for other routes of administration.

### 3.8. FT-IR Spectroscopy

**Figure 11:**
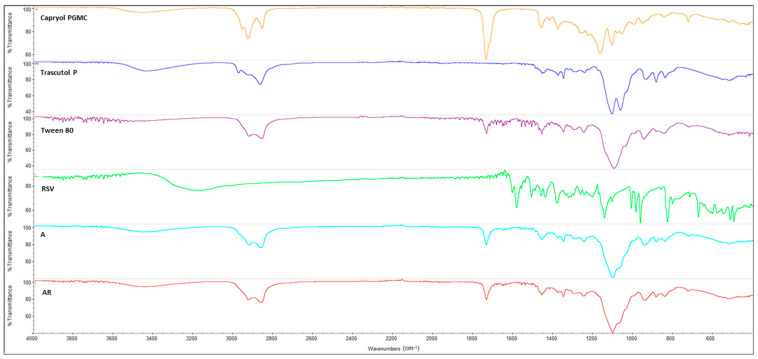
FTIR spectra of pure SNEDDS components and blank (A) and RSV-loaded (AR) nanocarriers.

**Figure 12:**
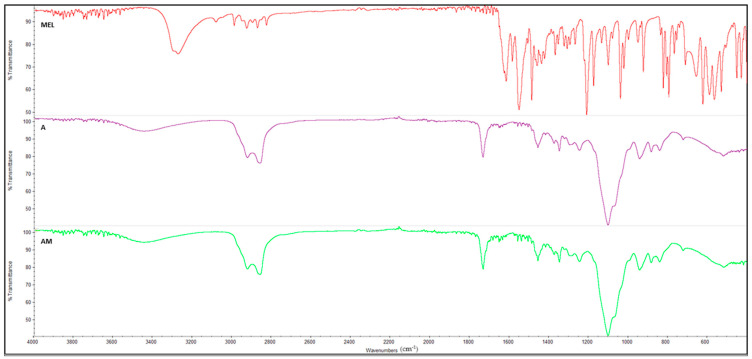
FTIR Spectra for MEL, blank SNEDDS (A) and MEL-loaded SNEDDS (AM).

To study any possible interaction between the drugs and components of the samples, FT-IR spectroscopic analysis was applied to the neat ingredients and to blank formulation A and formulation A loaded with RSV or MEL (AR and AM, respectively) (Figure 11 and Figure 12).

Figure 11 delineates the primary peaks of the used materials. They exhibit compatibility with each other, as evidenced by formulation A wherein the characteristic peaks of Capryol^®^ PGMC at 3447 cm^−1^ (O-H) and 1735 cm^−1^ (C=O) are distinctly visible, along with the aliphatic C-H carbon peaks between 2995–2856 cm^−1^, specific of Transcutol^®^ P and Tween^®^ 80. Moreover, the latter surfactant displayed a peak at approximately 1700 cm^−1^ (HOH bending) and another at 1096 cm^−1^ (C-O), both of which reappeared in the spectrum of formulation A. Despite slight shifts with minor significance, all components maintain their intrinsic characteristics within the blank formulation. The RSV-SNEDDS specimen exhibits a spectrum that was superimposable with the one of blank SNEDDS, encompassing all previously observed peaks. However, distinct RSV peaks, such as the one at 3177.63 cm^−1^ (corresponding to OH functional groups) and the peak around 1580 cm^−1^, are absent in the loaded formulation. This outcome, combined with the resemblance to the blank SNEDDS spectrum, suggests the effective encapsulation of RSV within the formulation [55]. Moreover, the absence of characteristic RSV peaks in the spectrum of AR formulation implies that the drug was situated inside the emulsion micelles rather than on their surface.

The NH stretching peak belonging to MEL at 3273.55 cm^−1^ was not observed in the spectrum of formulation AM (Figure 12), which completely overlaps with the unloaded formulation A. The peaks observed corresponded to those derived from the raw materials Capryol^®^ PGMC, Transcutol^®^ P, and Tween^®^ 80, already shown in Figure 11, also indicating, in this case, the complete entrapment of MEL within the micelles of the AM emulsion [56]. 

### 3.9. Mucoadhesion Study 

To assess the mucoadhesive properties of SNEDDS, the interaction with mucin was evaluated over a period of 3 h by measuring the absorbance of SNEDDS/mucin mixtures at 650 nm and any change in size and ZP values.

**Figure 13:**
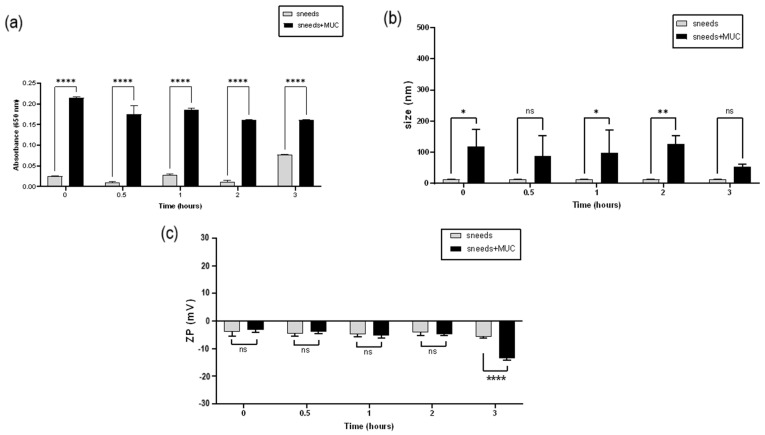
(**a**) Absorbance, (**b**) mean size and (**c**) ZP values of formulation A before (SNEDDS) and after different times of incubation with mucin dispersion in STF at 37 °C (SNEDDS+MUC). * *p* < 0.05, ** *p* < 0.01, **** *p* < 0.0001, ns = not significant.

As Figure 13 shows, there was a slight but significant interaction with mucin. Actually, the components of SNEDDS were not mucoadhesive materials and did not possess a net charge that could interact electrostatically with the negative charges of the protein. An increase in size and absorbance was apparent already after 15 min of contact with mucin and was maintained for the next 3 h. Mucin was absorbed into the lipid micelles, which led to the registered increase in size and, in turn, in turbidity. The increase in particle size was not followed by significant aggregation phenomena since PDI values remained ≤0.4. The turbidity measurement was found to be significant at all time points. However, the formulation maintained its transparency, as absorbance values were found to be ≤0.2, which is suited for ocular administration [57]. 

A stronger interaction could be proven by a change in the ZP value of the colloidal system once in contact with mucin [58]. In this case, it was evident that the interaction was not very strong and occurred noticeably after 3 h of contact. The use of ingredients with mucoadhesive potential or lipid materials with a positive charge could reinforce the interaction with mucin [59,60]. Mucoadhesive properties could improve the residence time of a formulation on the corneal surface. This is important since SNEDDS form very small globules, in the order of about 50 nm, after their dilution with the tear fluid. Such small particles can easily be carried away by the tear flow. By improving the interaction between the formulation and the mucin present on the ocular surface, the residence time can be ameliorated.

### 3.10. In Vitro Release in Simulated Ocular Environment

**Figure 14:**
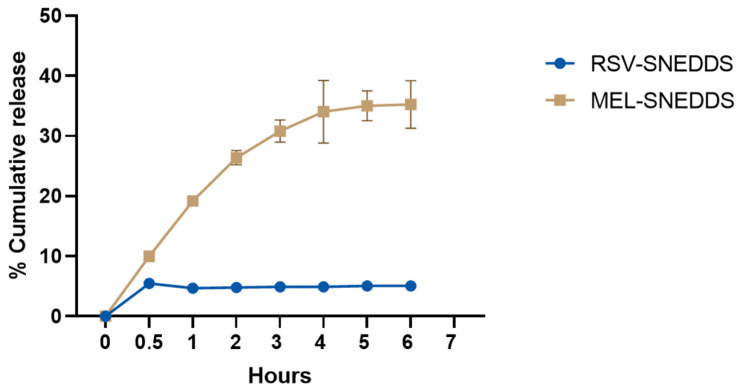
Cumulative release of RSV and MEL from AR and AM, respectively, in simulated ocular environment.

The cumulative release rate was calculated according to the released RSV and MEL compared to the total initial drug amounts. It is not simply to evaluate the effective release from a SNEDDS formulation. When SNEDDS comes in contact with the tear fluid, different entities are formed, including the free molecular state of the drug, the drug inside the nanoemulsion droplets, and the drug in the micellar solution [61]. In this case, both drugs seemed to have a good encapsulation within the systems, as suggested by the low release up to 6 h (Figure 14).

The release test was performed in STF at 35 °C, monitoring the drug release for 6 h. As can be observed, the very lipophilic RSV was retained by the lipid matrix and was released to a maximum of 5% in a constant manner. The outer medium also probably became saturated, giving a very low-release profile. Many studies prefer to use a medium added with a surfactant, e.g., Tween^®^ 80 at 2.5%, to raise the release up to 100% [62]. 

MEL followed a different pattern, its amphiphilic nature allowing a faster release over time, with a peak at 4 h with 35% of the released drug. The low and constant release of these drugs by SNEDDS formulations could be useful for a prolonged release. 

The release rate constant was estimated from the slope of the different curves, and regression values (R^2^) were obtained. Table 7 shows that the in vitro MEL release from SNEDDS was best described by both the Higuchi equation (R^2^ = 0.8685) and the Korsmeyer–Peppas equation (R^2^ = 0.8863). The former equation indicates that the drug release occurred via diffusion through the dispersed globules in a constant and controlled manner [63]. The second equation showed a diffusional release exponent parameter, indicated with *n*, which was 0.414. This suggests a quasi-Fickian diffusion profile (when *n* < 0.45) of MEL release [64]. Regarding the in vitro RSV release, this was best described by Korsmeyer–Peppas equation (R^2^ = 0.9736) with *n* = 0.084, indicating again a quasi-Fickian diffusion profile [64].

### 3.11. Short Time Exposure Test (STE)

**Figure 15:**
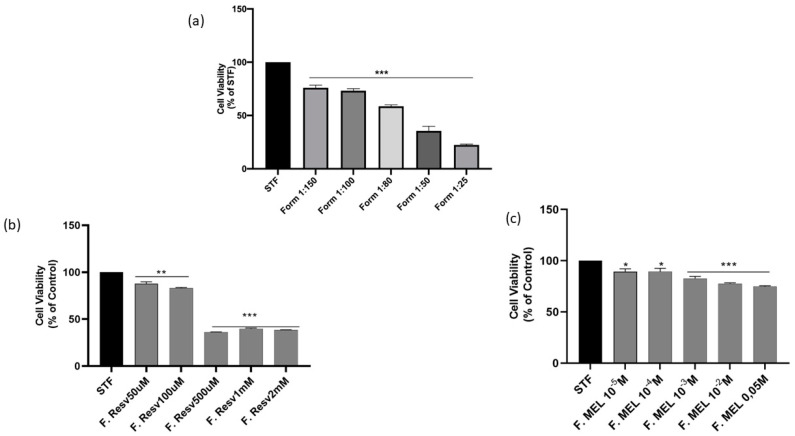
Effect of (**a**) blank formulation treatment on SIRC cell viability; (**b**) Cell viability of SIRC cultured in STF, representing the control group, or treated with different concentrations of AR (50 μM, 100 μM, 500 μM, 1 mM or 2 mM) for 5 min, or (**c**) with different concentrations of AM (10^−5^ M, 10^−4^ M, 10^−3^ M, 10^−2^ M, 0.05 M). * *p* < 0.05, ** *p* < 0.01, *** *p* < 0.001.

The STE test was used to assess the cytotoxicity of the formulated systems. This test, described by Takahashi et al., can be applied when formulations to be tested contain large percentages of surfactant(s). The test was performed on cells of the corneal epithelium, such as SIRCs, for a time of 5 min [23,24]. The evaluation of cytotoxicity was carried out by means of an MTT test. First, an analysis was made on the blank formulation (A) at different dilutions (Figure 15a). The dilution of 1:100 was thus chosen for further studies.

As Figure 15 shows, the concentrations tested on a 1:100 SNEDDS/STF dilution were 5 for the formulation with RSV (AR) and 5 for the formulation with MEL (AM). The choice of concentrations ranged from a minimum of the therapeutic drug concentration up to a maximum of 100 times the therapeutic drug concentration. RSV at concentrations of 50 μM and 100 μM was protective in retinal cells exposed to hypertension-derived damage through the regulation of a SIRT-1-related pathway [41]. MEL at a concentration of 10^−4^ M inhibited the activation of Müller cells (support for the retinal pigment epithelium) and the production of pro-inflammatory cytokines in a model of diabetic retinopathy through the upregulation of a SIRT-1 pathway [39,40]. It is well known that less than 5% of the dose reaches the back of the eye, so a concentrated formulation is preferable for better efficacy. All AM concentrations were compatible and non-toxic to SIRCs under the used test conditions. AR was cytotoxic at the highest concentrations, from 500 μM upwards [65,66].

## 4. Conclusions

The aim of this study was to design systems, using a statistical approach, with technological properties and characteristics suitable for topical ocular administration. The rationale of the study was to design SNEDDS for the delivery of SIRT-1 agonists in ocular degenerative diseases characterized by a downregulation of the enzyme. RSV and MEL were chosen as model drugs, both being well known for their effect in regulating the SIRT-1 pathway in inflammatory states of ocular tissues, in particular of the retina. 

Four formulations with four different surfactants (Tween^®^ 80, Tween^®^ 20, Cremophor^®^ EL, and Solutol^®^ HS15) were optimized and characterized in terms of mean size, PDI, ZP, pH, osmolarity, emulsion time, and transmittance (clearness). All these parameters were found to be compatible with a possible ocular administration. The SNEDDS formulations were loaded with RSV and MEL, giving excellent encapsulation results.

The formulation consisting of Capryol^®^ PGMC, Transcutol^®^ P, and Tween^®^ 80 was chosen for its higher physical stability for the subsequent studies. A slight mucoadhesive capacity was found after incubation with mucin in a simulated ocular environment. The formulations of RSV-SNEDDS and MEL-SNEDDS proved to be cytocompatible with cells of the corneal epithelium. 

The results of this work demonstrate that the use of a DoE approach can enable the optimization of formulations suitable for ocular administration that must encapsulate highly lipophilic drugs, increasing their apparent solubility in water. Further, in vivo studies are ongoing to evaluate the mechanism of diffusion of drugs towards the posterior eye segment and the pharmacological activity of the loaded nanocarriers.

## Figures and Tables

**Table 1:** Variables of Simplex Lattice Design for pseudo-ternary phase diagram.

Component	Units	Type	Minimum	Maximum
A (oil)	% (*w/w*)	Mixture	10	80
B (surfactant)	% (*w/w*)	Mixture	10	80
C (co-surfactant)	% (*w/w*)	Mixture	10	80
Constraints		Total (A + B + C) = 100		
Transmittance	T%	Response		

**Table 2:** Variables of the Design Space.

Factors	Name	Units	Type	Levels	
				Low	High
X_1_	Oil concn.	% (*w/w*)	Numeric	10	30
X_2_	Surfactant concn.	% (*w/w*)	Numeric	10	70
X_3_	Co-surfactant concn.	% (*w/w*)	Numeric	10	70
X_4_	Surfactant type		Categoric		Tween^®^ 80 Tween^®^ 20 Cremophor^®^ EL Solutol^®^ HS15
Constraints: X_1_ + X_2_ + X_3_ = 100					
Y_1_	Size	nm			
Y_2_	Time of emulsification	s			
Y_3_	Transmittance	%			

**Table 3:** Optimization criteria for SNEDDS production.

Factors and Responses		Goal	Lower Limit	Upper Limit
X_1_	Oil concn. % (*w/w*)	Minimize	10	30
X_2_	Surfactant concn. % (*w/w*)	Maximize	20	70
X_3_	Co-surfactant concn. % (*w/w*)	Minimize	20	70
X_4_	Type of surfactant	In range	Tween^®^ 80, Tween^®^ 20, Cremophor^®^ EL, Solutol^®^ HS15	
Y_1_	Mean particle size (nm)	Close to 20 nm	13.29	455.1
Y_2_	Time of emulsification (s)	Minimize	31	134
Y_3_	% Transmittance	Maximize	0.1	100

**Table 4:** Optimized formulation according to the desirability parameter.

Sample	Type of Oil	Oil Concn. %	Surfactant Concn. %	Co-Surfactant Concn. %	Desirability
A	Tween^®^ 80	15.041	55.181	28.211	0.886
B	Tween^®^ 20	15.456	52.471	30.133	0.723
C	Cremophor^®^ EL	14.351	58.025	23.358	0.761
D	Solutol^®^ HS15	14.351	58.025	23.358	0.868

**Table 5:** Experimental values of size, % transmittance, and time of emulsification for optimized SNEDDS.

Sample	Size (nm) ± SD	% Transmittance	Time of Emulsification (s)
A	13.26 ± 0.07	100	12.04
B	127.29 ± 1.12	88	12.18
C	11.29 ± 0.11	100	17.85
D	18.82 ± 0.41	100	15.76

**Table 6:** Temperature, size and PDI of different dilutions of SNEDDS/STF subjected to cloud point measurement.

Ratio SNEDDS/STF	Temperature (°C)	Size (nm) ± SD	PDI
1:10	47.2	210 ± 17.5	0.263 ± 0.090
1:50	60.5	201.1 ± 11.73	0.311 ± 0.049
1:100	70.0	120.1 ± 5.208	0.289 ± 0.024
1:200	79.9	443.8 ± 286	0.575 ± 0.601
1:300	82.2	1228.6 ± 16.09	0.561 ± 0.105

**Table 7:** Regression coefficient values (R^2^) for different release kinetic models obtained from the in vitro release profiles of loaded SNEDDS in STF. The calculations were made on the linear part of the release curves (from 0.5 h forward).

Sample	Zero Order	First Order	Higuchi	Hixson–Crowell	Korsmeyer–Peppas
MEL	0.7465	0.6142	0.8685	0.7692	0.8863
RSV	0.0116	0.0072	0.0456	0.0117	0.9736

## Data Availability

Data are contained within the article and supplementary materials.

## Supplementary Materials

The following supporting information can be downloaded at https://www.mdpi.com/article/10.3390/pharmaceutics16010125/s1, Supplementary Table S1. Physico-chemical properties of melatonin and resveratrol.
