# Supplementary material for: Formulating Resveratrol and Melatonin Self-Nanoemulsifying Drug Delivery Systems (SNEDDS) for Ocular Administration Using Design of Experiments
Source: Pharmaceutics. 2024 Jan 18;16(1):125. doi: 10.3390/pharmaceutics16010125 (PMC10819881; doi:10.3390/pharmaceutics16010125)
Supplement: Supplementary file 1 [file pharmaceutics-16-00125-s001.zip › pharmaceutics-2773642-supplementary.pdf]

Supplementary Table S1. Physico-chemical properties of melatonin and resveratrol.

Drug	Molecular mass (g/mol)	m.p. °C	Solubility	LogP	Form/ colour	Storage/ stability	Toxicity
**Melatonin**	232.30	114 to 124	Methanol (50 mg/ml)	1.65	White to slightly yellow powder	+2°C to +8°C Protect from light	None - Standard handling
**Trans-Resveratrol**	228.24	250-257	DMSO (50 mg/ml)	3.1	Off-white powder	+2°C to +8°C Protect from light	None - Standard handling
